# Harm reduction services as a point-of-entry to and source of end-of-life care and support for homeless and marginally housed persons who use alcohol and/or illicit drugs: a qualitative analysis

**DOI:** 10.1186/1471-2458-12-312

**Published:** 2012-05-17

**Authors:** Ryan McNeil, Manal Guirguis-Younger, Laura B Dilley, Tim D Aubry, Jeffrey Turnbull, Stephen W Hwang

**Affiliations:** 1 British Columbia Centre for Excellence in HIV/AIDS, St. Paul's Hospital, 608-1081 Burrard Street, Vancouver, BC, V6Z 1Y6, Canada; 2 Faculty of Human Sciences, Saint Paul University, Ottawa, ON, Canada; 3 Faculty of Education, Simon Fraser University, Surrey, BC, Canada; 4 School of Psychology, University of Ottawa, Ottawa, ON, Canada; 5 Department of Medicine, The Ottawa Hospital, Ottawa, ON, Canada; 6 Ottawa Inner City Health, Ottawa, ON, Canada; 7 Keenan Research Centre of the Li Ka Shing Knowledge Institute, St. Michael’s Hospital, Toronto, ON, Canada

## Abstract

**Background:**

Homeless and marginally housed persons who use alcohol and/or illicit drugs often have end-of-life care needs that go unmet due to barriers that they face to accessing end-of-life care services. Many homeless and marginally housed persons who use these substances must therefore rely upon alternate sources of end-of-life care and support. This article explores the role of harm reduction services in end-of-life care services delivery to homeless and marginally housed persons who use alcohol and/or illicit drugs.

**Methods:**

A qualitative case study design was used to explore end-of-life care services delivery to homeless and marginally housed persons in six Canadian cities. A key objective was to explore the role of harm reduction services. 54 health and social services professionals participated in semi-structured qualitative interviews. All participants reported that they provided care and support to this population at end-of-life.

**Results:**

Harm reduction services (e.g., syringe exchange programs, managed alcohol programs, etc.) were identified as a critical point-of-entry to and source of end-of-life care and support for homeless and marginally housed persons who use alcohol and/or illicit drugs. Where possible, harm reduction services facilitated referrals to end-of-life care services for this population. Harm reduction services also provided end-of-life care and support when members of this population were unable or unwilling to access end-of-life care services, thereby improving quality-of-life and increasing self-determination regarding place-of-death.

**Conclusions:**

While partnerships between harm reduction programs and end-of-life care services are identified as one way to improve access, it is noted that more comprehensive harm reduction services might be needed in end-of-life care settings if they are to engage this underserved population.

## Background

At any given moment, tens of thousands of people in Canada are homeless or marginally housed [1,2]—that is, live places unfit for human habitation (e.g., outdoors, vehicles, etc.) or temporary, transitional, or emergency accommodations (e.g., emergency shelters, hostels, etc.). Homeless and marginally housed persons have consistently reported levels of alcohol and/or illicit drug use many times higher than the stably housed population [3-7]. For example, a recent study of a large sample of homeless persons in Toronto found that 60% had a lifetime prevalence of regular illicit drug use and 40% reported active use of illicit drugs other than marijuana [3]. A cohort study of homeless and marginally housed youth in Vancouver reported that 41.1% had used drugs by injection [4]. Another study of homeless women in Vancouver noted that 82.4% of its sample regularly used substances, including illicit drugs (70.5%) and alcohol (37.8%) [5].

Homelessness and marginal housing are strongly correlated with a range of adverse health outcomes [8-10], including high levels of morbidity and mortality [11-13], that are frequently exacerbated by substance use. Injection drug use and non-injection use of crack cocaine and crystal methamphetamine are risk factors for HIV and Hepatitis C (HCV) infection [14-18], resulting in high infection rates among homeless and marginally housed persons who use these substances [18-20]. Furthermore, injection drug use and non-injection use of crack cocaine and crystal methamphetamine have been shown to accelerate HIV and HCV disease progression, leading to higher incidences of cirrhosis, hepatocellular carcinoma, opportunistic infections, and AIDS-related cancers among these populations [21-24]. Heavy alcohol consumption is similarly linked to HIV and HCV disease progression, resulting in similarly poor outcomes [25-27]. Homeless and marginally housed populations consequently suffer from high mortality rates and life expectancies at least a decade less than those of the general population [11-13].

Homeless and unstably housed persons who use alcohol and/or illicit drugs have complex end-of-life care needs, which are compounded by the challenges of everyday survival (i.e., securing food and shelter) [28-31]. In spite of a high level of need, this population faces many barriers to accessing the end-of-life care system [28,32]. While many populations in Canada report difficulties accessing end-of-life care services (e.g., Aboriginal peoples and people living in rural areas [33-35]), the realities of homelessness and drug and/or alcohol use pose particular challenges. End-of-life care services have been largely developed based on assumptions (i.e. individuals are housed, supported by family and friends, and/or have resources to pay for supplementary care) that do not reflect the experience of this population. Homeless and marginally housed persons who use alcohol and/or illicit drugs lack access to end-of-life care services where these services are delivered via home care or pay-for-service facilities. Furthermore, end-of-life care services providers often enforce rules and regulations (e.g. anti-drug policies) that exclude this population from accessing care [32]. Although several low-threshold hospices have implemented harm reduction policies (i.e.: permitting onsite prescribed alcohol use, providing injecting equipment and permitting off-site illicit drug use), this end-of-life care service delivery model has not been widely implemented.

Many homeless and marginally housed persons who use drugs and/or alcohol presumably rely on alternate sources of care and support at end-of-life. While researchers have increasingly turned their attention toward the end-of-life care needs of this population [28-32], they have not explored alternate sources of end-of-life care and support for this population. For example, what services do homeless and unstably housed persons who use alcohol and/or illicit drugs turn to for end-of-life care and support? What level of support and care are these services able to provide? And also, what role do these services play in facilitating access to more comprehensive end-of-life care?

In Canada, as in many other countries, harm reduction programming is a central component of public health strategies. Harm reduction emphasizes interventions that minimize the drug and health harms associated with alcohol and/or drug use (e.g., overdose, infectious diseases, etc.) while not requiring abstinence as a condition for accessing services [36]. A growing body of research has noted that harm reduction services are one of the principal and often only sources of medical care and referrals for homeless and marginally housed persons who use alcohol and/or illicit drugs in Canada [37-42]. However, this research has overlooked any role that these programs play in facilitating access to end-of-life care and support for this population. This article explores how harm reduction services act as a point-of-entry to and source of end-of-life care and support for this population based on qualitative interviews with key informants in six Canadian cities. Although data collection was limited to a country with universal healthcare insurance, findings from this study provide insights that are likely applicable to other jurisdictions where harm reduction programs are involved in the delivery of health and social services to homeless and marginally housed persons.

## Methods

### Study design

We used a qualitative case study design to explore end-of-life care services delivery to homeless and unstably housed persons with problematic use of alcohol and/or illicit drugs [43], with an emphasis on points-of-entry to and sources of end-of-life care and support. We conducted qualitative interviews with health and social services professionals (e.g., emergency shelter directors, physicians, nurses, etc.) between February 2007 and August 2008 in which we explored this topic. Given the lack of previous research in this area, qualitative interviews were deemed to be the best means through which to generate preliminary insights into factors that shape sources of end-of-life care for this population. We originally intended to also conduct interviews with homeless and unstably housed persons who use alcohol and/or illicit drugs that had been diagnosed as being terminally ill at the primary site of this study (i.e., Ottawa), but determined this was not feasible after conducting preliminary interviews with these participants (n = 5). We ultimately decided that we could not guarantee that the data would be of sufficient quality due to poor recall of events and inconsistency in the reporting of these events. We were also concerned that many participants would be unable to provide informed consent due to cognitive deficits (e.g., HIV-related dementia, impaired cognitive function due to stroke, etc.) and interviews would place an undue burden on participants with a high severity of illness. In spite of this limitation, we felt that interviews with key informants (i.e., health and social services professions who provide end-of-life care and support to homeless populations) would allow us to generate the best possible insights into the needs of this population.

### Participants & recruitment

Key informants involved in the delivery of health and end-of-life care services to homeless and unstably housed persons in Halifax, Ottawa, Hamilton, Toronto, Thunder Bay, and Winnipeg were invited to participate in this study. An advisory committee made up of regional experts (i.e., senior health and social services professionals who provide services to homeless persons), as well as existing relationships with homeless services organizations in Ottawa and Toronto, helped us to identify key informants in those cities. Key informants in Halifax, Hamilton, Thunder Bay, and Winnipeg were identified through a scoping review of health services for homeless persons in those cities.

Seventy-three potential participants were sent a letter or email by the lead author providing an overview of the study and inviting them to participate. Fifty-four individuals (74%) agreed to participate in this study, including physicians (6), nurse practitioners (2), nurses (16), social workers (5), emergency shelter or supportive housing executive directors and/or senior managers (9), harm reduction specialists (5), outreach workers (7), and personal support workers (4). Many participants worked in harm reduction-based settings, such as low-threshold hospices (15), managed alcohol programs (8), and syringe and/or safer crack use kit distribution programs (8), and the remainder of participants reported regular contact with these services.

### Data collection

Semi-structured interviews were conducted with participants at their place of employment or an alternate location of their choosing. Participants were asked to discuss a range of topics related to end-of-life care services delivery to homeless and marginally housed persons who use alcohol and/or illicit drugs, including points-of-entry and sources of end-of-life care and support. Interviews ranged in length from 45 to 120 minutes, although the majority of interviews were approximately one hour in length. Interviews were audio recorded with the consent of participants and transcribed verbatim by research assistants. Each transcript was reviewed by the lead author while listening to the audio file to verify the accuracy of the transcript and make any necessary corrections.

### Data analysis

The goal of analysis for this article was to explore how homeless persons who use alcohol and/or illicit drugs gain access to end-o-life care services, as well as non-traditional sources of end-of-life care and support. Transcripts were imported into qualitative analysis software (NVivo v.8) to facilitate coding [44]. A preliminary set of two categories extracted from the interview guide (e.g. points-of-entry to end-of-life care services and sources of end-of-life care and support) was used to provide an initial framework for the analysis. We concurrently coded the transcripts by drawing upon constant comparative methods, whereby emerging sub-categories were recorded and explicated by means of constant comparison to the data [45,46]. We determined early in analysis that harm reduction services served as a key point-of-entry and source of end-of-life care and support for homeless and marginally housed persons who use alcohol and/or illicit drugs. We adjusted the coding framework to account for the outreach (i.e. drop-in and outreach syringe exchange and distribution) and residential programs (i.e., services, such as managed alcohol programs and low threshold emergency shelters, where clients are permitted to be intoxicated or actively use) available in these cities. We discussed and further revised the coding framework during subsequent meetings until the final themes and sub-themes were established. Once these final categories were established, the lead author independently recoded sections of the data to verify the validity of these categories.

### Ethics

This study received approval from the institutional research ethics committees at Saint Paul University and the University of British Columbia. Informed consent was obtained prior to the interviews and participants retained a duplicate copy of the informed consent protocol for their records. In order to maintain participant anonymity and confidentiality, where participants are quoted directly, they are identified by their professional role.

## Results

Participants identified many barriers to end-of-life care services for homeless and marginally housed persons who use alcohol and/or illicit drugs, which are reviewed in-depth elsewhere [32]. This article focuses instead on the role harm reduction services play in addressing gaps in end-of-life care services by providing care and support for homeless and marginally housed persons who use alcohol and/or illicit drugs. Eight themes are grouped into three domains to shed light on this dynamic: first, how harm reduction programs act as a point-of-entry to end-of-life care services; second, how harm reduction outreach programs serve as a source of end-of-life support; and, finally, how residential harm reduction programs serve as a source of end-of-life care and support. Direct quotations are used throughout to illustrate these themes.

### Harm reduction services as a point-of-entry to end-of-life care services

#### Increasing engagement with population

Participants reported that, while multiple social and structural barriers restricted access to end-of-life care services for homeless and marginally housed persons who use alcohol and/or illicit drugs (e.g. lack of affordability, abstinence-only policies, etc.), one of the principal barriers was an overall lack of engagement with health and social care services. They noted that delivering services via harm reduction programs helped them to establish contact and build trusting relationships with this population. Participants felt that, by using a non-judgmental harm reduction approach, they were able to better engage with this population. As one participant noted:

"You have to earn it. You have to show that you want to do something for them. I’m going to make sure that it gets done and that it’s followed up. You have to be respectful and treat people with the same kind of treatment that you would want. I’m often very informal. I use humour but, most of all, I think that you earn it. It's often word of mouth. One client will say, “Listen, you can trust her." (Harm Reduction Outreach Worker)"

"Individuals come in through the needle exchange program and they come in and talk. Over time, you build a sense of confidence and then become more conversational about what’s happening and what their needs are. It takes time for people to be willing to trust or to think it is worthwhile to trust. (Harm Reduction Outreach Worker)"

Participants strongly felt that these relationships allowed them to better facilitate referrals to end-of-life care services for this population. Several physicians acknowledged the critical role that harm reduction services played in facilitating access to end-of-life care services for this population. For example:

"It’s the outreach worker that will do the vast majority of the work. It’s thanks to these guys that I’m able to see the clients. The client has trust with the team and then I become a part of the team so they’re able to trust me. (Physician)"

#### Engaging with clients over time

Participants reported that, since many homeless and marginally housed persons who use alcohol and/or illicit drugs regularly accessed harm reduction services, they were able to maintain frequent contact with this population. Participants articulated how this long-term engagement mediated access to end-of-life care services. Many participants noted that, because harm reduction programs provided ongoing and often the only care and support for this population, they were positioned to monitor changes in health status. Consequently, they were positioned to facilitate referrals to end-of-life care services when necessary. For example:

"We have a lot of the guys that been here [harm reduction program] for six years now. They’re all getting older and you can tell that the alcohol is hitting them harder. At one point, their health needs are just too much just for us but there’s nowhere for them to go unless they’re really actually sick and can go to the end-of-life care provider. (Nurse)"

"He’s there every day. He’s getting sicker. He’s not housed. Three or four of these clients since I’ve started working here have been recognized by the workers at [harm reduction program]. They know to call us and that we’ll follow through with helping with appointments and referrals to the [end-of-life care provider]. (Nurse)"

#### Maintaining relationships with end-of-life care providers

Participants felt that it was important for harm reduction programs and end-of-life care providers to maintain relationships to facilitate access to end-of-life care services for homeless and marginally housed persons who use alcohol and/or illicit drugs. Many participants who worked for harm reduction programs indicated that they sought out opportunities to formally link with end-of-life care providers in their communities. These participants believed that these links with end-of-life care providers not only served to facilitate referrals, but also allowed them to generally advocate for end-of-life care services for this population. One participant remarked:

"We are now part of a community palliative care committee. That committee looks at the services that are available and what’s needed—the gaps that exist for homeless and under-housed in terms of end-of-life care. We might recognize that someone is very ill so it is a matter of getting them to a physician and then making an application to move them into a palliative care facility. (Nurse)"

Many participants who worked in end-of-life care settings also acknowledged the importance of these links. These participants reported that links with harm reduction programs helped them gain access to a population that they would not otherwise be able to provide end-of-life care to. For example:

"People who are living on the street…it’s much harder to access them. They don’t come to us and they don’t go anywhere for help until they’re so sick that they’re picked up by an ambulance. I’ll get calls from street nurses. Somebody will say, "I’ve got somebody that I think is going to need to come to you at some point.” (Hospice Administrator)"

### Harm reduction outreach services as a source of end-of-life care

#### Providing end-of-life support to clients unable to access services

Participants reported that harm reduction outreach programs were often one of the only sources of end-of-life support available to this population. Participants acknowledged that, while these programs lacked the resources to provide end-of-life care (e.g. pain and symptom management, counselling, etc.), they were able to provide a range of support services via these programs. Many of these support services (e.g. personal support, housing assistance, etc.) were intended to help homeless and marginally housed persons who use alcohol and/or illicit drugs meet their most basic needs (e.g. shelter, food, etc.) at end-of-life and thereby minimize to the greatest extent possible the hardships associated with poverty and homelessness. For example:

"We had one fellow that died but he had no place to live. He wasn’t welcome at the local shelter because of some of his behaviours. He was sitting out there in his wheelchair on the corner ready to take on all comers and the police brought him to our office. A co-worker had a big office that had a couch in it and the police would bring him in with a bag of McDonalds. He would lie on the couch for the day. At the end of the day, we all had homes to go to and we couldn’t let him stay there. We would find a place and sometimes we would find someone willing to put him in a hotel overnight. It would be four-thirty on a Friday afternoon and I would be searching and scrounging and finally find one loophole and get him some place for the weekend. (Harm Reduction Outreach Worker)"

Participants reported that unstably housed persons (e.g. living in single room occupancy hotels or apartments not fit for human habitation) with substance use challenges also received end-of-life support via harm reduction outreach programs. They noted that they provided end-of-life support services (e.g.: personal care, emotional support, etc.) to this population as part of or in addition to the regular outreach services. Participants identified several reasons why this population received end-of-life support from these programs, such as poor access to home care services and comfort with harm reduction outreach workers. As one participant noted:

"We had a few clients that were old time clients that were HIV-positive and turned into AIDS. There was a place down on the South end where a lot of the addicts live. We had support staff that would go down and help with this person. We’ll make home visits because they feel more comfortable with us because they’ve been with us for so long. (Harm Outreach Worker)"

"When I first started working here, there was a client who was dying of AIDS and [an outreach worker] used to go down every day and of sit with him for a while…There was another guy dying from hepatitis C complications—liver cirrhosis—and [the outreach worker] would go sit with him, talk to him, help him out, you know. Clean him and wash him, things like that. (Harm reduction outreach worker)"

#### Providing end-of-life support to clients who wish to ‘die at home’

Participants indicated that, when end-of-life care services were available, some unstably housed persons who used substances refused referrals to end-of-life care providers, preferring instead to die-at-home (e.g. single room occupancy hotels or apartments not fit for human habitation). Participants reported that such persons indicated to them that the benefits of end-of-life care services (i.e., supportive care, pain and symptom management, etc.) were outweighed by the drawbacks, notably disruptions in living environment (e.g. peer support networks, etc.) and lack of self-determination of place-of-death. Participants who worked at harm reduction outreach programs expressed that they were committed to supporting clients who wished to die at home. As a result, they reported that they provided and facilitated a range of end-of-life support services for this population that were outside of the scope of their everyday responsibilities. For example:

"We had one fellow that was an injection drug user. He was HIV-positive for many years and continuously used and drank. He was in and out of hospital. He got to the point where he knew he was dying and he wanted to die with his [single room occupancy hotel] friends. We were able to work around that. We had nurses from here going in. We had doctors going in. It was home until he finally did die. (Harm reduction outreach worker)"

"There was an individual I’d worked with for a number of years…He lived [in an apartment in the west end] and he died of Hepatitis C and HIV there. I would go over and see him and we’d talk and I’d smudge him [i.e. perform an Aboriginal purification ceremony]. It was just a matter of being there for him on a daily basis. (Harm reduction outreach worker)"

### Residential harm reduction programs as a source of end-of-life care

#### Providing culturally-competent care

Participants reported that residential harm reduction programs (i.e. managed alcohol programs and low barrier emergency shelters) served as a key source of end-of-life care services for homeless persons who use substances at end-of-life. Many participants noted that the delivery of end-of-life care services in these settings was an extension of the range of other clinical and support services delivered onsite (e.g. medication assistance, nursing care, etc.) and responded to the natural progression of health needs experienced by their clients (e.g. HCV, HIV/AIDS, etc.). Participants acknowledged that, while unable to provide services equal to those provided in end-of-life care settings, they had greater cultural competence in providing care for this population. Participants strongly felt that they were better able to provide care that responded to the unique needs of this population and acknowledged their lived experiences. As one participant observed:

"Staff members are not trained in palliative care but they have many years of experience working with people with heavy substance use, heavy alcohol use, difficult behaviours and mental health problems. This is the group that they’ve chosen to work with. It’s not like five percent of the people that they work with. It’s everybody they work with. (Physician)"

#### Providing end-of-life care for clients in a home setting

Participants acknowledged that many clients identified residential harm reduction programs as home and expressed a strong desire to die there. One of the key reasons for this was that many clients of these programs had been long-term residents of these programs and established strong relationships with other residents and staff. Many of the participants in this study articulated how strong relationships with residents motivated them to support these clients at end-of-life. For example:

"The guys know that we’ll keep them here as long as possible and that we’ll try to increase nursing hours. We’ll try to do anything to keep them here because they are family. It’s like they want to die in their home. (Nurse)"

"We would discuss their wishes in terms of where they would like to spend their last weeks or months and their options would basically fall into two categories. It would be either arranging something at the hospital or spending their last bit of time in this program. Certainly, we wouldn’t be able to provide the same level of care that they might receive in the hospital but we might still be more desirable—passing away at home because the program environment had indeed become their home and their community. (Physician)"

#### Implications of end-of-life care and support for regular services

Participants acknowledged that delivering end-of-life care services via residential harm reduction programs impacted the delivery of regular services to program clients. One of the key implications noted by participants was that they had to divert limited resources to providing end-of-life care to clients. Several participants reported that in spite of their best intentions it became necessary to refer some of these clients to end-of-life care settings when it became clear that they would become overextended. One participant noted:

"It got to the point with cirrhosis of the liver that he had problems going to the bathroom. He couldn’t sleep on the overnight and was afraid to sleep because he thought he was not going to wake up. Staff members had to basically spend twenty four hours with this individual. That is when we realized we had nine other residents. Staff members were saying that we really want to support this client but it’s impossible for us. At that point, we said, ‘Okay, we really need to make a referral.’ (Harm reduction specialist)"

Many participants also articulated how they learned from these experiences in order to be better equipped to provide care to subsequent clients who were dying. For example:

"We kind of got caught off guard because our first client that got sick was quite young and experienced profound liver failure, extremely fast. He had to go to the hospital. He didn’t want to but he had to. We didn’t have any nursing support in place. We didn’t have equipment. We didn’t have the drugs. He had to go to the hospital to die. It set us into our planning stage for the next event—getting equipment, nurses, drugs, and all these things in place for the next person. (Nurse Manager)"

## Discussion

Over the course of this study, participants articulated how harm reduction services act as an alternate source of end-of-life care and support for homeless and marginally housed persons who use alcohol and/or illicit drugs. Participants highlighted how establishing trusting relationships and regularly engaging with clients facilitated referrals to end-of-life care services. Previous research has similarly noted that harm reduction services (e.g. syringe exchange programs, supervised injection facilities, etc.) are key points-of-referral to health care services for substance-using populations [37-42], but only recently has attention turned to the health care interactions that facilitate these referrals [47-49]. For example, in a recent study of client perspectives of a syringe exchange program in a mid-sized Canadian city, MacNeil and Pauly [47] noted that adopting a non-judgmental approach led to the trusting relationships between clients and staff as well as linkages to other healthcare services. While the findings presented in this article further outline the importance of trust in mediating access to healthcare services—in this case, end-of-life care services, closer scrutiny of the characteristics of these relationships is needed to identify *how* they improve engagement with this population.

Participants also identified harm reduction programs as an alternate and often the only source of end-of-life care and support available to homeless and marginally housed persons who use alcohol and/or illicit drugs. For example, participants reported that harm reduction outreach programs provided a range of end-of-life support services (e.g. personal support, housing assistance, etc.) for unstably housed persons who were unable or unwilling to access end-of-life care services as a result of social and structural barriers to these services. While participants felt that they improved clients’ quality of life by providing these basic support services, they acknowledged that they could not provide care equal to that provided in end-of-life care settings. In light of these limitations, there remains a need to develop interventions that minimize to the greatest extent possible barriers this population faces to end-of-life care services. Fostering partnerships between harm reduction outreach programs and end-of-life care teams is one approach that might improve access to end-of-life care for this population [50,51]. Harding et al. [51] have previously noted that partnerships between community health programs (including substance use programs) and end-of-life care teams are critical to enhancing access and equity in end-of-life care services for underserved populations.

Our findings also suggest that homeless or unstably housed persons who use substances might refuse referrals to end-of-life care services because they wish to die at “home” (i.e., residential harm reduction programs, single room occupancy hotels, or apartments unfit for human habitation) supported by harm reduction outreach workers. Participants pointed out that they provided end-of-life care and support for this population because they were motivated to support clients who wished to die at home (or had no alternative). Although researchers have increasingly explored the end-of-life care needs of this population (28-32), they have largely overlooked any concerns related to *place-of-death* for homeless and unstably housed persons, in general, and homeless and marginally housed persons who use alcohol and/or illicit drugs, in particular. Our findings suggest that many homeless and marginally housed persons who use alcohol and/or illicit drugs might wish to die-at-home for many of the same reasons their stably housed counterparts wish to (e.g. comfort, familiarity, presence of loved ones, etc.) [52]. And yet, precisely because this population typically lacks reliable caregivers and/or home-based end-of-life care services, they run the risk of dying alone, anonymously, and with unmet care needs. While harm reduction programs play a vital role in minimizing these adverse outcomes, action is clearly needed to address multiple social and structural inequities (e.g. availability of affordable housing, home care services, etc.) that constrain the ability of this population to exercise agency in regards to place of death.

Finally, our findings echo those of previous studies reporting that harm reduction programs serve as an alternate healthcare delivery system for homeless persons who use substances [53,54]. Some of the main advantages of delivering healthcare services via harm reduction programs include increased responsiveness to the population’s needs and flexibility, as well as promoting dignity and respect for persons who use substances [53,54]. However, while alternate healthcare services delivery via harm reduction programs improves access for homeless and marginally housed persons who use alcohol and/or illicit drugs, it also leads to separate but not equal healthcare services for this population [54]. In light of this concern, more comprehensive strategies in addition to those outlined above are needed to reduce or eliminate the barriers that substance-using populations face to accessing end-of-life care services.

Integrating more comprehensive harm reduction approaches (e.g. supervised drug consumption services) into end-of-life care services represents one way to potentially improve access and equity in end-of-life care services for this population [32]. A growing body of research suggests that supervised drug consumption services are an effective strategy for increasing health access [55,56], minimizing accidental overdoses [55,57,58], and encouraging safer drug use practices [59,60] among illicit drug users. Further research is needed to determine whether these and other benefits extend to other healthcare settings adopting this approach. Partnering organizations with expertise providing these services with the end-of-life care system is worth exploring to facilitate their expansion into end-of-life care settings.

We acknowledge that harm reduction programs have faced community opposition [61-63], which might restrict their integration into these settings, yet several health and end-of-life care service providers have nonetheless successfully implemented harm reduction approaches. For example, the Dr. Peter Centre, a community-based health facility in Vancouver, Canada that provides clinical and support services to persons living with HIV/AIDS including end-of-life care, has integrated supervised injection services into its programming since 2002. An evaluation of the impact of comprehensive harm reduction services in these settings might yield further insights into how harm reduction programming might be integrated into end-of-life care.

### Limitations

This study has several limitations that should be noted. Our findings have limited generalizability to regions where health care services are organized differently (e.g. lack of universal health insurance) and/or drug policy prohibits harm reduction services or limits their role in health care services delivery to this population. While this articles draws on interviews with a range of experienced health and social care professionals who work with homeless and marginally housed persons who use alcohol and/or illicit drugs, it does not report on the first hand experiences of this population. Additional research is needed to explore the specific concerns and experiences of this population**.** Finally, this qualitative study was exploratory in nature. The insights generated will help to inform further research.

## Conclusion

In Canada, as elsewhere [28], the end-of-life care system is largely inaccessible to homeless and marginally housed persons who use alcohol and/or illicit drugs. This study explored how harm reduction programs (e.g., syringe exchange programs, managed alcohol programs, etc.) have emerged as an important point-of-referral to and vital alternate source of end-of-life care for this population. Importantly, harm reduction programs were perceived as a means to provide culturally-competent care and increase self-determination regarding place-of-death. Notwithstanding the benefits of this approach, action is clearly needed to increase access *and* equity in the end-of-life care system for this population. This study identified several ways that this may be achieved, namely increased collaboration between the public health and end-of-life care systems and adoption of harm reduction approaches in end-of-life care settings.

## Competing interests

The authors declare that they have no competing interests.

## Authors’ contributions

All authors contributed to the conceptualization of this study. RM and MGY conducted the interviews. RM, MGY, and LBD conducted the analysis. RM wrote the first draft of the manuscript. All authors contributed to the critical revision of the manuscript and approved the final version.

## Pre-publication history

The pre-publication history for this paper can be accessed here:

http://www.biomedcentral.com/1471-2458/12/312/prepub
